# Understanding the clinical reasoning processes involved in the management of multimorbidity in an ambulatory setting: study protocol of a stimulated recall research

**DOI:** 10.1186/s12909-020-02459-w

**Published:** 2021-01-07

**Authors:** M.-C. Audétat, S. Cairo Notari, J. Sader, C. Ritz, T. Fassier, J. M. Sommer, M. Nendaz, N. Caire-Fon

**Affiliations:** 1 Primary Care Institut (iuMFE), Faculty of Medicine, University of Geneva, CMU 5-6, Rue Michel-Servet 1, 1211 Geneva, Switzerland; 2 Unit of Development and Research in Medical Education (UDREM), Faculty of Medicine, University of Geneva, Geneva, Switzerland; 3 Department of Family and Emergency Medicine, Faculty of Medicine, Université de Montréal, Montreal, Canada; 4 Faculty of Psychology and Educational Sciences, University of Geneva, Geneva, Switzerland; 5 Division of Internal Medicine for the elderly, University Hospitals of Geneva, Geneva, Switzerland

**Keywords:** Study protocol, Clinical reasoning, Multimorbidity, Primary care, Ambulatory setting, Qualitative study, Stimulated recall

## Abstract

**Background:**

Primary care physicians are at the very heart of managing patients suffering from multimorbidity. However, several studies have highlighted that some physicians feel ill-equipped to manage these kinds of complex clinical situations. Few studies are available on the clinical reasoning processes at play during the long-term management and follow-up of patients suffering from multimorbidity. This study aims to contribute to a better understanding on how the clinical reasoning of primary care physicians is affected during follow-up consultations with these patients.

**Methods:**

A qualitative research project based on semi-structured interviews with primary care physicians in an ambulatory setting will be carried out, using the video stimulated recall interview method. Participants will be filmed in their work environment during a standard consultation with a patient suffering from multimorbidity using a “button camera” (small camera) which will be pinned to their white coat. The recording will be used in a following semi-structured interview with physicians and the research team to instigate a stimulated recall. Stimulated recall is a research method that allows the investigation of cognitive processes by inviting participants to recall their concurrent thinking during an event when prompted by a video sequence recall. During this interview, participants will be prompted by different video sequence and asked to discuss them; the aim will be to encourage them to make their clinical reasoning processes explicit. Fifteen to twenty interviews are planned to reach data saturation. The interviews will be transcribed verbatim and data will be analysed according to a standard content analysis, using deductive and inductive approaches.

**Conclusion:**

Study results will contribute to the scientific community’s overall understanding of clinical reasoning. This will subsequently allow future generation of primary care physicians to have access to more adequate trainings to manage patients suffering from multimorbidity in their practice. As a result, this will improve the quality of the patient’s care and treatments.

**Supplementary Information:**

The online version contains supplementary material available at 10.1186/s12909-020-02459-w.

## Background

Multimorbidity, commonly defined as the co-occurrence of at least two chronic diseases [1, 2], is widespread and increasing in the population worldwide [3–6]. Patients suffering from multimorbidity represent more than 50% of the general practitioners’ (GPs) practice in most countries [7–9]. As Starfield has stated, one of the major current challenges to primary care revolves around recognizing and managing multimorbidity [10]. Thus, there is a strong and growing interest in how to provide quality healthcare services for these patients [11–15].

Reports have put forward the added-value of a strong primary health care system recognizing the role of primary care as a pivotal organization for ensuring proper use of professional skills in the management of patients suffering from multimorbidity [3]. Based on theoretical models such as the Chronic Care Model, initiatives targeting improvements in the care of these patients have been implemented worldwide [15, 16]. Primary care physicians being at the heart of the practice, puts them in an ideal position to provide the care these patients need [17, 18], as this involves patient-centered care, coordination and collaboration between several healthcare professionals [19, 20], as well as better knowledge of the various chronic diseases and their possible interactions [21].

However, the challenges in this realm of care are numerous. Results from a systematic review authored by Sinnott et al. [22] showed that the difficulties encountered by GPs may be classified into four domains: 1) disorganization and fragmentation of healthcare; 2) inadequacy of guidelines and evidence-based medicine; 3) challenges in delivering patient-centered care; and 4) barriers to shared decision making [22].

Clinical reasoning processes are intrinsically involved in all these domains of specific difficulties. Clinical reasoning is usually defined as the thought and decision-making processes with the aim to reach a problem resolution [23]. Research in cognitive psychology in the last decades have contributed to a better understanding of these processes [24, 25]. But most of the research has focused on the clinical reasoning at play while reaching a diagnosis. This can be easily explained, as “reaching the correct diagnosis” is seen as the main goal of clinical problem solving in medicine [26].

Nevertheless, taking care of patients suffering from chronic diseases requires that clinical reasoning continues beyond their diagnosis and also includes choices regarding treatment, follow-up visits, further testing, etc. [26]. An emerging literature relates to these reasoning processes involved during longitudinal follow-up, calling them *management reasoning* [27]. In a previous explorative study (under review), we showed that rather than achieving a diagnosis, the main goal of these clinical reasoning processes is the search for a balance between the evidence-based care options, the patient’s priorities and trying to withhold their quality of life, through the consideration and prioritization of several possibilities. This may imply for the GPs to accept lower levels of disease control than recommended by the guidelines. These results are congruent with the ones of Sinnott and al., who suggests the concept of “satisficing”: clinicians provide the care they consider to be satisfactory and sufficient for a given patient in his or her particular context [28].

Despite their relevance in the primary care clinical setting, clinical reasoning processes involved during the management of chronic care remains poorly described in the literature. It is therefore necessary to untangle the different processes at play and to better understand how they articulate with one another during the longitudinal care of patients suffering from multimorbidity [20].

The effort to understand these clinical reasoning processes may also support clinical training and supervision. Indeed, despite the high prevalence of multimorbidity in primary care, physicians might feel ill-equipped to manage the challenges that come along with these clinical situations. A US survey evaluated physicians’ perceptions of the adequacy of their chronic illness care training: most of them reported that, with regard to the demands of their current practice, they had not received adequate training [29]. Medical schools and residency programs may need to adapt curricula to train and prepare physicians to treat the growing number of patients suffering from multimorbidity. Calls have been made for improving clinical and general practice training in order to address the challenges of multimorbidity [12, 30, 31], as dealing with complexity is part of the competencies trainees have to acquire [32].

The ambulatory care setting is of paramount importance for the training of physicians [33, 34]. This clinical setting allows learners to engage in authentic professional tasks and problem-solving and confronts them with the complexity of chronic patients, thus providing a very fertile learning environment [35–41]. This is particularly true when it comes to developing the necessary competencies for the management of multimorbidity [21, 42]. Supervision plays a key role in learning to reason clinically in that context [43]. It can foster learning through role-modelling, feedback, and encourage the articulation of one’s thinking [44–46]. But since physicians have often developed their clinical expertise in a tacit and informal way [3, 47, 48], it may be difficult for them to supervise and teach their trainees in a targeted and specific manner. Further research is thus needed to better understand how clinical reasoning processes are at play during the long-term follow-up care of patients suffering from multimorbidity, and how we can foster them during supervision of trainees in the ambulatory clinical setting.

## Methods

### Aim

Our study aims to understand how primary care physicians think and what are the clinical reasoning processes at play during the follow-up consultations with patients suffering from multimorbidity.

### Design

A qualitative approach appears most suited to the study aim and the research question we are seeking to answer, as we plan to shed some light on processes which are not accurately understood in practice and teaching [49, 50]. Individual encounters with primary care physicians working in ambulatory settings will be carried out using the video stimulated recall interview (SRI) method [51]. In SRI, participants view a video sequence of their behaviour (in our case, a clinical encounter) and are invited by the researcher to reflect on their decision-making processes. The SRI serves the participant as a help to recall what was in his or her mind at the moment of the action or discussion seen in the video [51].

This research method produces both insightful and useful data for examining and understanding the cognitive processes participants use in a specific interaction and the way they use them. It has been used for a long time in social sciences [52–54]. This technique is considered the most powerful tool in retrospective studies on clinical reasoning in authentic settings, compared with free recall or audio-assisted recall, because the video provides interviewees with rich and vivid cues to explain their thinking during the activity [55, 56]. SRI also enables subjects to recall more events, to live a greater experiential immersion, and to recollect and describe up to 4 times more detail compared with free recall [57].

In our study, SRI will unfold as follows: (a) primary care physicians will be filmed during an encounter with one of their patients suffering from multimorbidity; (b) researchers will watch the recording, identifying units of meanings or specific moments they want to discuss with the physician during the SRI; (c) physicians will then be shown video recordings of their work. The playback will be interrupted in respect to the research question at certain moments in order to give the participants an opportunity to explain their clinical reasoning and thoughts about the just seen sequence. Participants will be invited to stop the playback whenever they want to add something according to the instructions given.

There are many issues when using a video camera to record someone’s activity. To make the video recording as easy as possible, and cause as little disturbance as possible during the clinical encounter with the patient, we plan to record physicians’ activity from an “own-point-of-view” perspective, using a micro camera. This technique helps participants retrospectively articulate their thought processes by minimizing self-consciousness, by maximizing their psychological immersion in the activity preceding the interview, and by triggering memories of these cognitive processes [57].

### Sample

Participants will be recruited using the Exponential Snowball Sampling method [58]. This method is suitable when it is difficult to recruit participants (as it is often the case for physicians in private practice). One member of the researcher team, a primary care physician, will thus recruit three participants among her colleagues. Each participant will then be asked to suggest three other colleagues corresponding to our inclusion criteria (see below). Researchers will ensure that the set of participants is sufficiently representative of the community (not only hospital setting, not the same ambulatory practice, not only clinical teachers). Sampling will continue until data saturation.

The recruitment process will be based on the following inclusion criteria: (a) primary care physicians working in ambulatory settings (private practice or in hospital) at least 3 days a week in the area of Geneva (Switzerland); (b) not specialized in the follow-up of only one kind of disease; (c) and recommended by peers. Our list of inclusion criteria deliberately does not take into account years of clinical experience because the validity of this criterion for research purpose has been called into question, as quality of care may not be correlated with years of experience [59]. This risk seems to be even greater with regard to clinical reasoning [60].

An information and consent form will state the study objectives and the fact that participation is voluntary and unpaid (a compensation in the form of vouchers will be given). All participants will sign a written consent form that specifically authorizes the video recording of their work activity and the audio recording of the interview. Written consent will also be required from patients to be video recorded and to authorize the use of their clinical situations in order to understand physician’s thinking.

According to Swiss legislation, this project does not fall within the scope of the LRH (Loi relative à la recherche sur l’être humain) article 2. A waiver to enter a full ethical review was granted by the Research Ethics Committee of Geneva (CCER.GE.CH).

### Data collection

Physicians will be asked to select their next patient with multimorbidity (i.e. ≥ 2 chronic diseases) their first morning consultation of the day, 4 weeks after they accept to participate in the study. These criteria for patients’ selection limit potential selection bias.

The scheduled consultation should be at least 30 min to obtain sufficiently rich data. Oral consent from the patient will be required before he or she comes to the physician’s office for the consultation. Prior to the start of the consultation, the patient will sign a consent form. An ad hoc self-reported questionnaire will be used to ask the physician some basic information about the clinical situation being discussed.

A member of the research team will come to the practice 30 min before the beginning of the consultation to set up the equipment, i.e., the installation of the camera the physician will wear during the consultation in order to film the patient.

No team member will be present during the consultation with the patient, but a researcher will return at the end of the consultation to pick up the study material and allow the physician to continue his or her work. The same day, after having selected the video sequences for the SRI, members of the research team will meet the physician for the interview during about 1 h. Participants will be asked to render their clinical reasoning processes explicit and to provide meaning to their actions. Based on the reasoning processes described by the participant, members of the research team will also address the key issues emerging from the exploratory research (under review), following a semi-structured guide (see Supplementary file 1).

The interviews will be held in series of 5 until no new information related to our study aim emerge from the analysis, indicating saturation of data [61]. Fifteen to 20 interviews lasting about 60 min are planned for this purpose. Data collection and analysis will take place iteratively. Each interview will be audio-recorded, then transcribed verbatim and anonymized for qualitative analysis.

### Data storage and management

Data (i.e. video recordings, transcripts, and questionnaires) will be safely stored in a certified repository at the University of Geneva for long term preservation and curation under the responsibility of the main investigator. All data will be anonymized and labeled in a uniform way to ensure interoperability.

### Data analysis

Transcripts will be imported into Atlas.ti. The use of this qualitative analysis software will facilitate the creation of codes, manual encoding, and storage and recovery of segments of verbatim reports attached to each code.

Data will be analysed according to a standard content analysis, using deductive and inductive approaches [49, 62]. The deductive approach will be based on the clinical reasoning model developed by Charlin et al. [63]. This clinical reasoning model constitutes an explicit graphical representation of the multifaceted processes of clinical reasoning and could be of value as a framework to enlighten the processes at play. Inductive approach will allow us to consider the themes directly emerging from the data.

### International collaboration

We also plan to conduct this research in the primary care context of the province of Quebec, in collaboration with the family care department of the Université de Montréal, Canada. This will allow us to compare our data and enrich our analyses.

## Pilot study

To evaluate the feasibility and suitability of the study, a pilot was conducted in 2019 at two GP’s offices. This valuable experience has led to a reorganization of certain technical aspects, allowing the improvement of the study protocol. Four main changes were made.

First, the camera positioning was adapted. Our initial choice was a micro camera mounted at the physician’s eye level, but we decided to use a little “button camera” that is placed like a pin on the doctor’s white coat and is much more discrete. This pilot reinforced that this new positioning of the camera did not interfere with the physician-patient clinical encounter or undermine the physicians’ ability to recall their clinical reasoning during SRI.

Second, contrary to what was initially foreseen, two researchers with different trainings (one medical doctor and one psychologist), and not just one of them, will be present during the interviews of the participants. This gives more depth and allows the physicians to bounce back on different ideas which enriched the interaction and thus the collected data.

Third, given the complexity of the topic at hand, we have decided to start the interview by asking the participants to describe with a metaphor how they perceive themselves while managing these patients. The use of a metaphor offers GPs a gateway towards their implicit reasoning processes, as showed by several studies [64, 65].

Four, we adapted our sampling method. We first tried to recruit our participants by sending an invitation to join the study to primary care clinical teachers working in private practice. As the response rate to our invitation was very low, we chose to recruit participants using the Exponential Snowball Sampling method [58].

Beyond these adjustments, the pilot study allowed us to validate the relevance and acceptability of our research design and setting. Carrying a camera does not seem disruptive of the processes being studied, and interviewees have no difficulty extracting the relevant information from the video. Moreover, clinicians were all welcoming and enjoyed the interview process as it prompted them to become aware of their clinical reasoning and make it explicit, which they all found an enriching experience.

## Discussion

Primary care physicians are at the very heart of managing patients with multiple chronic diseases. At least two useful developments are expected from this study: scientific advances and practical impacts.

Firstly, study results will contribute to the scientific community’s overall understanding of clinical reasoning as it is used in the context of patients with multiple chronic conditions by primary care physicians during the long-term follow-up care of their patients in ambulatory settings. Facing the challenge of meeting the complex needs of these patients requires to deeply involve patients and their relatives, as well as different healthcare providers in an interprofessional approach: in this perspective, many issues are still to be considered in order to implement shared decision-making processes as well as a more collaborative reasoning between healthcare professionals [20].

Secondly, our results would enable clinicians to be more conscious of the richness and quality of their own clinical reasoning processes used during multimorbidity management and, in turn, would allow them to develop an explicit role model and thereby better facilitate their students’ learning processes during supervision sessions.

From a practice perspective, the dissemination of the results among primary care physicians, as well as in the context of continuous professional development and faculty development, will contribute directly to clinicians and learners’ competency development. As university centres for research and teaching in primary care, we plan to develop courses for primary care physicians based on these results so as to improve the skills to teach the decision-making process and clinical reasoning used for efficient management of chronic multiple conditions so as to improve the future management of these patients.

## Supplementary Information

**Additional file 1 Supplementary file 1.** Semi-structured interview guide.

## Data Availability

Not applicable.
