# Supplementary material for: Understanding the clinical reasoning processes involved in the management of multimorbidity in an ambulatory setting: study protocol of a stimulated recall research
Source: BMC Med Educ. 2021 Jan 7;21:31. doi: 10.1186/s12909-020-02459-w (PMC7792096; doi:10.1186/s12909-020-02459-w)
Supplement: Supplementary file 1 — Additional file 1 Supplementary file 1. Semi-structured interview guide. [file 12909_2020_2459_MOESM1_ESM.docx]

***Supplementary file 1***

Semi-structured interview guide

| 1. | How do physicians plan (i.e. organise in advance in their head) the clinical consultation? What are the benefits to this organisation? |
| --- | --- |
| 2. | How do physicians prioritize which problems to put in the forefront for the given consultation? |
| 3. | What are the physicians’ global aims of the care? Are these aims what motivates their choice and interventions of the different problems at hand? |
| 4. | How do physicians manage to combine their own aims of the consultation and keeping them in alignment with the aims of the patients? Is there any collaborative reasoning with the patient or close relatives? |
| 5. | How do physicians investigate a problem? Do they use standard scripts? How do they use guidelines? Do they make links to other diseases or problems? Do they tend to develop an analytical or more intuitive reasoning? |
| 6. | How do physicians share their reasoning with peers or third party? How does the reasoning of peers may or not influence their own clinical reasoning? Is it a collaborative reasoning? How do they coordinate with other specialists or healthcare professionals? |
| 7. | Which clinical reasoning processes are used by physicians at the end of the consultation? What are the aims of these processes? |
| 8. | What actions physicians plan to take as a result of the consultation? What clinicians’ decisions or action plans (e.g. contact a specialist, collaboration with a nurse, discussion about a particular issue with the patient at the next visit)? |
